# Changes in the hyoid bone, tongue, and oropharyngeal airway space after mandibular setback surgery evaluated by cone-beam computed tomography

**DOI:** 10.1186/s40902-020-00271-6

**Published:** 2020-08-12

**Authors:** Seon-Hye Kim, Sung-Kwon Choi

**Affiliations:** 1 Department of Orthodontics, School of Dentistry, Wonkwang University, Iksan, Korea; 2 College of dentistry, Graduate School of Wonkwang University, Iksan, Korea

**Keywords:** Mandibular setback surgery, Hyoid bone, Tongue, Oropharyngeal airway space, Three-dimensional changes

## Abstract

**Background:**

Mandibular setback surgery can change the position of the mandible which improves occlusion and facial profile. Surgical movement of the mandible affects the base of the tongue, hyoid bone, and associated tissues, resulting in changes in the pharyngeal airway space. The aim of this study was to analyze the 3-dimensional (3D) changes in the hyoid bone and tongue positions and oropharyngeal airway space after mandibular setback surgery.

**Methods:**

A total of 30 pairs of cone-beam computed tomography (CBCT) images taken before and 1 month after surgery were analyzed by measuring changes in the hyoid bone and tongue positions and oropharyngeal airway space. The CBCT images were reoriented using InVivo 5.3 software (Anatomage, San Jose, USA) and landmarks were assigned to establish coordinates in a three-dimensional plane. The mean age of the patients was 21.7 years and the mean amount of mandibular setback was 5.94 mm measured from the B-point.

**Results:**

The hyoid bone showed significant posterior and inferior displacement (*P* < 0.001, *P* < 0.001, respectively). Significant superior and posterior movements of the tongue were observed (*P* < 0.05, *P* < 0.05, respectively). Regarding the velopharyngeal and glossopharyngeal spaces, there were significant reductions in the volume and minimal cross-sectional area (*P* < 0.001). The anteroposterior and transverse widths of the minimal cross-sectional area were decreased (*P* < 0.001, *P* < 0.001, respectively). In addition, the amount of mandibular setback positively correlated with the amount of posterior and inferior movement of the hyoid bone (*P* < 0.05, *P* < 0.05, respectively).

**Conclusion:**

There were significant changes in the hyoid bone, tongue, and airway space after mandibular setback surgery.

## Background

Orthognathic surgery for skeletal deformity is the standard of care for improving esthetics, occlusal relationship and stomatognathic function. Mandibular setback surgery is usually the treatment of choice for mandibular prognathism [1]. The posterior movement of the mandible induces positional changes of the hyoid bone and tongue base. This posterior shift of the tongue base causes a posterior extension of the soft palate and creates an increase in soft palate contact length, which can consequently decrease the pharyngeal airway space (PAS) [2, 3].

Obstructive sleep apnea (OSA) is a common sleep disorder caused by airway collapse at multiple levels of the upper airway, resulting in airway obstruction [4]. Several cases of postsurgical OSA have been reported by some authors [5, 6] since the 1980s. Demetriades et al. [7] reported that the incidence of mild-to-moderate obstructive sleep apnea syndrome was higher in patients with a mandibular setback of 5 mm or more than in the group with setback of less than 5 mm.

Recently, the percentage of class III patients being treated with bimaxillary surgery has been increasing [8]. However, isolated mandibular setback surgery is still the first choice in many cases with mandibular prognathism. Therefore, clinicians need to understand the postoperative changes that follow mandibular setback surgery. Unfortunately, there has been a lack of research assessing the changes of the hyoid bone, tongue, and oropharyngeal airway space in patients treated solely with mandibular setback surgery.

Previously, cephalometric analysis was used to evaluate the effects of mandibular setback surgery on the pharyngeal airway space, and while this analytical method is useful for measuring airway dimensions on the sagittal plane, it does not provide a full-scale view of the upper airway [9]. More recently, cone-beam computed tomographic (CBCT) images have been found to be useful in diagnostic and morphometric analysis of the airway dimensions [10, 11], soft tissue, and surrounding airway space [12, 13]. However, according to an American Association of Orthodontists white paper [14], CBCT does not provide information on neuromuscular tone or actual function of the airway.

Despite these limitations, CBCT provides more useful information than that available with 2-dimensional radiographs [15]. Therefore, the aim of this study was to use CBCT images to evaluate 3-dimensional (3D) changes of the hyoid bone, tongue, and oropharyngeal airway space in patients with prognathic mandible who had only undergone mandibular setback surgery.

## Materials and methods

### Subjects and CBCT image acquisition

This retrospective study was approved by the institutional review board (IRB) of the Wonkwang University (WKDIRB201810-01, WKUDHIRB-201810-03). A total of 30 patients (mean age, 21.7 years; range, 18.0–35.4 years; 14 males, 16 females) were selected from Wonkwang University Dental Hospital in Iksan and Daejeon to be included in the study. The inclusion criteria were (1) patients who had only undergone mandibular setback surgery and had dental and skeletal class III malocclusion showing an anterior overjet of 0 or less, and an ANB angle of 0 or less, and (2) patients who had undergone presurgical orthodontic treatment. The exclusion criteria were (1) patients who had undergone bimaxillary surgery, (2) patients with severe facial asymmetry, (3) patients with history of previous orthognathic surgery, (4) patients with history of trauma, (5) patients with cleft lip and/or palate, and (6) patients with any syndromes related to the orofacial region. All patients had been treated with bilateral sagittal split ramus osteotomy (BSSRO) and had received rigid fixation. Nineteen of them had had genioplasty at the same time.

Preoperative CBCT scans of each patient were taken about 1 month before surgery (T0). The postoperative CBCT examinations were performed about 1 month after surgery (T1) to check on postoperative swelling. The primary outcome variables were changes in the hyoid bone position and oropharyngeal airway space evaluated at 1 month before and after surgery. When taking CBCT images, the CBCT equipment (Alphard-3030; ASAHI Roentgen IND, Kyoto, Japan) was set at 80 kVp and 5.0 mA, and the image accusation time was 17 s, with a voxel size of 0.39 mm in the cranial mode. All patients were seated upright with the Frankfort horizontal plane parallel to the floor and their heads were fixed with a chincup and ear rod. They were asked to hold their breath after the end of expiration, without swallowing [16]. After the images were taken, they were imported as digital imaging and communications in medicine (DICOM) files with INFINITT PACS software program (INFINITT healthcare Co., Ltd, Seoul, Korea).

### Measurements

The DICOM files were reconstructed as 3D images with InVivo 5.3 software (Anatomage, San Jose, USA). All of the CBCT images were reoriented parallel to the Frankfort horizontal plane (FH plane) constructed by the right orbitale and both sides of the porion, perpendicular to the FH plane, passing through nasion and sella (midsagittal plane) and perpendicular to the other reference planes passing through the nasion (frontal plane).

Landmarks and reference planes for the measurements are described in Table 1. The landmarks were traced and their coordinates were expressed in three-dimensions. After tracing both T0 and T1 CBCT images of a single subject, they were superimposed, using the anterior cranial base as a reference and the same software program (InVivo; Anatomage, San Jose, USA) described above.

**Table 1:** Landmarks for measurements used in this study

Landmarks	Definition
N (nasion)	Point of contact between frontal bone and suture between 2 halves of nasal bones
S (sella)	Midpoint of the sella turcica
Po (porion)	Most superior point of external auditory meatus
Or (orbitale)	Lowest point on infraorbital margin of each orbit
B (B-point)	Most concave point on mandibular symphysis
Hb (hyoid bone point)	Most anterosuperior point of the hyoid bone
TT (tongue tip)	Most anterior point of the tongue
U1 (maxillary incisor)	Incisal edge of the maxillary central incisor

**Fig. 1:**
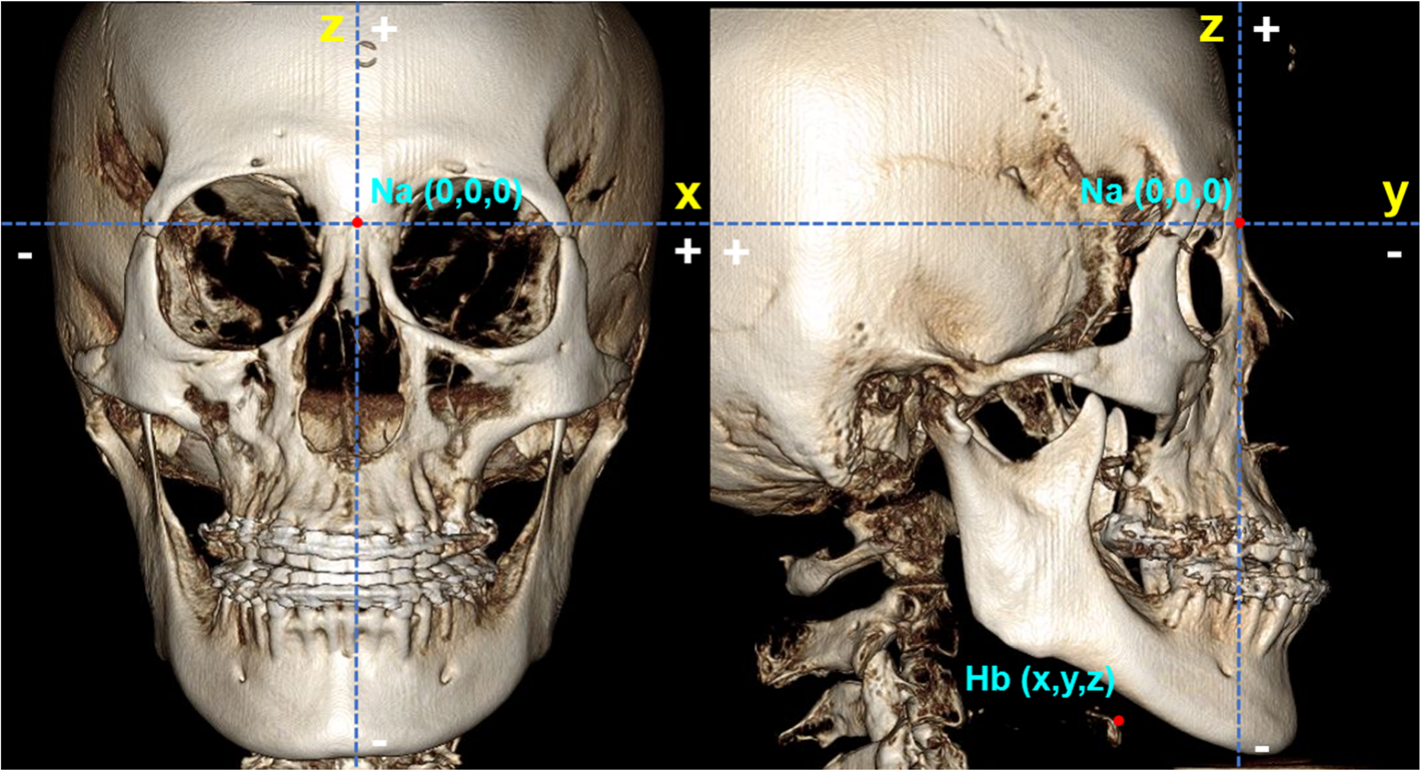
The plus and minus values along the three axes used in this study. The coordinates of the landmarks were assigned according to nasion (0, 0, 0). For the *x*-axis, landmarks on the left side are (+) values and the right side (−) values. For the *y*-axis, landmarks located posterior to the nasion are (+) values and anterior to the nasion (−) values. For the *z*-axis, landmarks located superior to the nasion are (+) values and inferior to the nasion (−) values. The coordinates of the Hb point are identified in T0 and T1 CBCT images

Results were measured by calculating the difference between T0 and T1 for each coordinate of the landmarks. The *x*-axis is in the transverse dimension, the *y*-axis is in the anteroposterior dimension, and the *z*-axis is in the vertical dimension. The plus and minus directions are indicated along the three axes (Fig. 1).

The amount of setback of the mandible was measured from the calculated value of the *y* coordinates of the B-point. The average mandibular setback was 5.94 ± 2.43 mm. Using the method stated above, the displacement of the hyoid bone could be measured quantitatively in three-dimensions.

#### Measurement of the hyoid bone position

Measurement of the hyoid bone position was done by calculating the difference of each coordinate of the Hb point between T0 and T1. The Hb point was the most anterosuperior point of the hyoid bone. The coordinates of the Hb were assigned according to nasion (0, 0, 0).

#### Measurement of the tongue dimensions and position

**Fig. 2:**
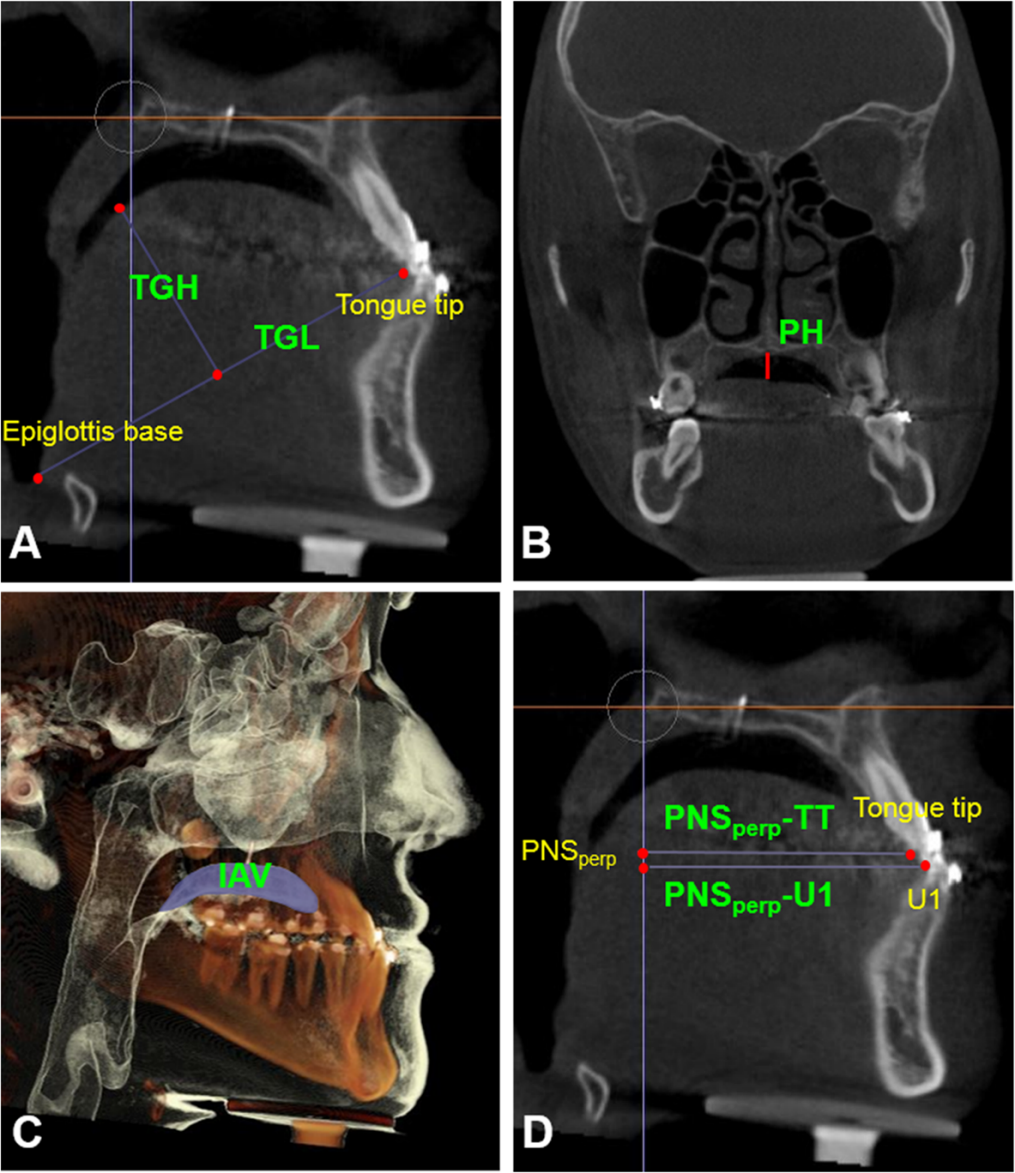
Tongue dimensions and position measurements. **a** Tongue length (TGL) (mm), the distance from epiglottis base and tongue tip; tongue height (TGH) (mm), the length of the vertical bisector from the dorsal tongue surface to a line connecting between the epiglottis base and tongue tip. **b** Palatal height (PH) (mm), the distance between the tongue’s highest point and the palate in the coronal view. **c** Intraoral airway volume (IAV) (mm^3^), the empty space between the tongue and palatal section. **d** PNS_perp_-TT/PNS_perp_-U1, the ratio of the distance from TT and U1 to a PNS perpendicular line to the FH plane

Measurements taken to assess the tongue dimensions were tongue length and height (mm). Measurements taken to assess the tongue position were palatal height (mm), intraoral airway volume (mm^3^), and PNS_perp_-TT/PNS_perp_-U1. Palatal height and intraoral airway volume indicate the vertical position of the tongue. PNS_perp_-TT/PNS_perp_-U1 indicates the sagittal position of the tongue. Definitions of tongue dimensions and positional measurements are described in Table 2 and Fig. 2.

**Table 2:** Definitions of tongue dimension and position and oropharyngeal airway space measurements used in this study

Measurements	Definition
Tongue length (TGL) (mm)	Length of tongue between epiglottis base and tongue tip (epiglottis base, the point located at the intersection of the epiglottis and the base of tongue)
Tongue height (TGH) (mm)	The length of the vertical bisector from the dorsal tongue surface to a line connecting between epiglottis base and tongue tip
Palatal height (PH) (mm)	The distance between the tongue’s highest point and the palate
Intraoral airway volume (IAV) (mm^3^)	The empty intraoral space between the tongue and palatal section
PNS_perp_-TT/PNS_perp_-U1	The ratio of the distance from TT and U1 to a PNS perpendicular line to the FH plane
Velopharynx (VP)	The area between a plane parallel to the Frankfort horizontal passing through posterior nasal spine (PNS) and a plane parallel to the Frankfort horizontal passing through the end of the soft palate
Glossopharynx (GP)	The area bounded superiorly by the inferior border of velopharynx and inferiorly by a plane parallel to the Frankfort horizontal passing through the tip of the epiglottis

#### Measurements of the oropharyngeal airway space

Measurements taken to assess the oropharyngeal airway space were the volume (mm^3^) and the cross-sectional measurements of the velopharynx and glossopharynx. Cross-sectional measurements include the minimal cross-sectional area (MCA) (mm^2^), anteroposterior width (mm), and transverse width (mm) of the minimal cross-sectional segment. “Minimal cross-sectional area” means the most constricted part of the airway.

**Fig. 3:**
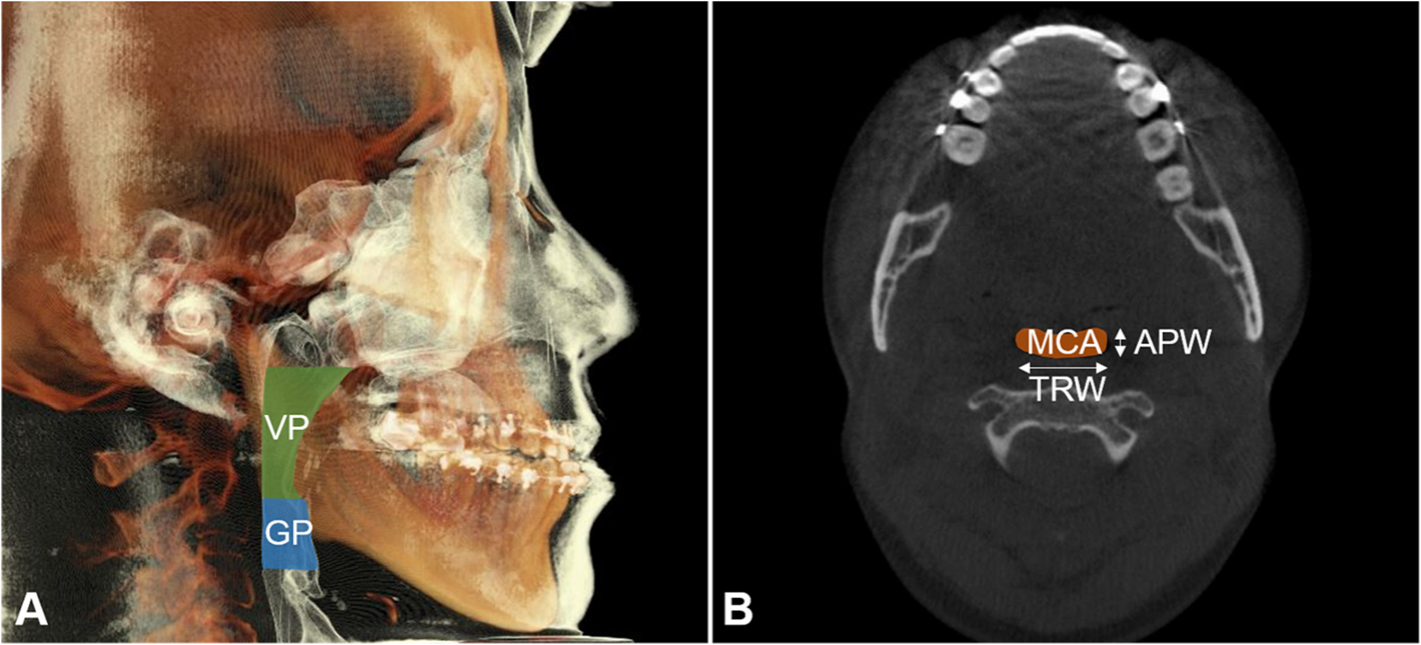
Oropharyngeal airway space measurements. **a** Segmentation of velopharynx (VP) and glossopharynx (GP) volume (mm^3^). **b** Minimal cross-sectional area (MCA) (mm^2^), anteroposterior width (APW) of MCA (mm), and transverse width (TRW) of MCA (mm)

The velopharynx was defined as the area between a plane parallel to the Frankfort horizontal passing through posterior nasal spine (PNS) and a plane parallel to the Frankfort horizontal passing through the end of the soft palate. The glossopharynx was defined as the airway bounded superiorly by the inferior border of velopharynx and inferiorly by a plane parallel to the Frankfort horizontal passing through the tip of the epiglottis (Table 2 and Fig. 3).

The volume of each segment of the airway space was calculated similarly to the study by Kim et al. [17]. The threshold values were set for a range of − 1,000 to − 300 Hounsfield units to eliminate imaging artifacts and to refine the selected airway region. The software automatically calculated the volume of the velopharynx and glossopharynx in cubic millimeters.

Cross-sectional measurements including MCA, anteroposterior width, and transverse width were also calculated. After the MCA was determined, the anteroposterior and transverse widths were measured.

### Statistical analysis

The sample size was determined based on the change in *y* coordinate of the hyoid bone from a small-scale pilot study of 7 subjects. The assumed mean difference was 1.78 ± 3.11 mm. The measurement of anteroposterior position of hyoid bone was adopted because it might be closely related to the posterior movement of mandible. It was determined that 26 subjects would be needed to provide a power of 0.82 and two-tailed alpha value of 0.05. A power analysis using G*Power (version 3.1.9.2: Franz Faul, Christian-Albrechts-Universitat, Kiel, Germany) was used to calculate the sample size required for this study.

The tracing and superimposition process of all CBCT images was done by 1 examiner (S.H.K.) to minimize measurement error. Ten randomly selected CBCT images were traced and superimposed again after a 2-week interval to evaluate the intraclass reliability. The intraclass correlation coefficient was from 0.827 to 0.902, which is considered to be excellent for intraclass reliability testing. To assess interexaminer agreement, the interclass correlation coefficient was also calculated by having a second examiner (S.K.C.) trace and superimpose for only pre-treatment records (T1) of 10 patients and it ranged from 0.804 to 0.914.

Paired *t* tests were used to evaluate significant differences in the mean value of the hyoid bone position measurements, tongue dimensions and position measurements, and the oropharyngeal airway space measurements from before surgery (T0) to after surgery (T1). A Pearson correlation test was done to identify the correlation between the amount of setback and the changes of the hyoid bone, tongue, and oropharyngeal airway space measurements. All statistical analysis was performed with the SPSS version 12.0 software (SPSS Inc., Chicago, IL, USA).

## Results

The investigated patients had a moderate dental and skeletal class III malocclusion (overjet of − 1 mm to − 4 mm, bilateral Angle class III molar relationship, and ANB cephalometric measurement of − 1° to − 4°).

### Changes in *x*, *y*, and *z* coordinates of the hyoid bone

There were significant differences in the changes in the *y* and *z* coordinates (*P* < 0.001), but not in the *x* coordinate (*P* > 0.05).

The *x* coordinate decreased by − 0.04 ± 1.55 mm (*P* > 0.05). The *y* coordinate significantly increased by 3.30 ± 2.90 mm (*P* < 0.001). The *z* coordinate significantly decreased by − 0.99 ± 4.16 mm (*P* < 0.001) (Table 3).

**Table 3:** Displacement of hyoid bone in *X*-, *Y*-, and *Z*-axes before and after surgery (mm)

	T0	T1	ΔT1 − T0	*P* value
X(Rt(−)-Lt(+))	− 0.37 ± 3.51	− 0.42 ± 3.75	− 0.04 ± 1.55	0.879
Y(Ant(−)-Post(+))	50.46 ± 9.13	53.76 ± 9.34	3.30 ± 2.90	0.000***
Z(Inf(−)-Sup(+))	− 116.09 ± 9.26	− 121.08 ± 9.78	− 4.99 ± 4.16	0.000***

Data are presented as mean ± standard deviation. Paired *t* tests were performed to determine significant differences in displacement of the hyoid bone in *x*-, *y*-, and *z*-axes between T0 and T1. *T0* before surgery, *T1* 1 month after surgery, *Rt* right, *Lt* left, *Ant* anterior, *Post* posterior, *Inf* inferior, *Sup* superior

*P < 0.05; **P < 0.01; ***P < 0.001

### Comparison of changes in tongue dimensions and positional measurements

There was no significant difference in the changes in tongue length and tongue height (*P* > 0.05). However, there were significant differences in the positional measurements (*P <* 0.05).

The palatal height and intraoral airway volume significantly decreased (− 2.73 ± 5.19 mm, − 6028.30 ± 10659.29 mm^3^, respectively, *P <* 0.05). PNS_perp_-TT/PNS_perp_-U1 significantly decreased by − 0.07 ± 0.09 between T0 and T1 (*P <* 0.05) (Table 4).

**Table 4:** Changes in tongue dimension and position before (T0) and after (T1) surgery

Variable	T0	T1	ΔT1 − T0	*P* value
TGL (mm)	68.82 ± 5.21	69.96 ± 5.97	1.13 ± 3.08	0.054
TGH (mm)	31.01 ± 4.73	31.71 ± 3.82	0.71 ± 3.17	0.231
Palatal height (mm)	7.84 ± 5.80	5.11 ± 5.21	− 2.73 ± 5.19	0.007**
Intraoral airway volume (mm^3^)	14043.97 ± 10009.12	8015.67 ± 9450.49	− 6028.30 ± 10659.29	0.004**
PNS_perp_-TT/PNS_perp_-U1	0.88 ± 0.08	0.82 ± 0.09	− 0.07 ± 0.09	0.001**

Data are presented as mean ± standard deviation. Paired *t* tests were performed to determine significant differences in tongue dimension and position between T0 and T1. *T0* before surgery, *T1* 1 month after surgery, *TGL* tongue length, *TGH* tongue height

*P < 0.05; **P < 0.01; ***P < 0.001

### Comparison of changes in oropharyngeal airway space measurements

The volume of the velopharynx and glossopharynx significantly decreased between T0 and T1 (− 3589.67 ± 3054.52, − 4268.43 ± 5020.01 mm^3^, respectively, *P* < 0.001). The minimal cross-sectional area of both the velopharynx and glossopharynx showed statistically significant decreases (− 103.85 ± 107.41, − 106.28 ± 103.08 mm^2^, respectively, *P* < 0.001). Also, significant decreases were noted in the mean anteroposterior width of the velopharynx (− 2.53 ± 2.57 mm, *P* < 0.001) and glossopharynx (− 2.18 ± 2.23 mm, *P* < 0.001), and in the mean transverse width of the velopharynx (− 5.65 ± 6.64 mm, *P* < 0.001) and glossopharynx (− 5.09 ± 7.41 mm, *P* < 0.01) (Table 5).

**Table 5:** Changes in pharyngeal airway space before (T0) and after (T1) surgery

Variable	T0	T1	ΔT1 − T0	*P* value
VP volume (mm^3^)	16777.20 ± 4689.63	13187.53 ± 4748.41	− 3589.67 ± 3054.52	0.000***
GP volume (mm^3^)	22952.70 ± 6983.11	18684.27 ± 6576.47	− 4268.43 ± 5020.01	0.000***
VP MCA (mm^2^)	325.43 ± 113.15	221.57 ± 131.54	− 103.85 ± 107.41	0.000***
GP MCA (mm^2^)	325.52 ± 121.94	219.24 ± 132.74	− 106.28 ± 103.08	0.000***
APW at VP (mm)	12.29 ± 3.21	9.77 ± 3.43	− 2.53 ± 2.57	0.000***
TRW at VP (mm)	27.75 ± 4.92	22.10 ± 8.17	− 5.65 ± 6.64	0.000***
APW at GP (mm)	12.36 ± 3.89	10.18 ± 3.75	− 2.18 ± 2.23	0.000***
TRW at GP (mm)	26.65 ± 5.44	21.56 ± 8.09	− 5.09 ± 7.41	0.001**

Data are presented as mean ± standard deviation. Paired *t* tests were performed to determine significant differences in oropharyngeal airway space between T0 and T1. *T0* before surgery, *T1* 1 month after surgery, *VP* velopharynx, *GP* glossopharynx, *MCA* minimal cross-sectional area, *APW* anteroposterior width, *TRW* transverse width

*P < 0.05; **P < 0.01; ***P < 0.001

### Correlation between the amount of mandibular setback and the changes in the hyoid bone, tongue, and oropharyngeal airway space

There were significant, positive correlations between the amount of mandibular setback and the amount of posterior and inferior movement of the hyoid bone with a coefficient of 0.410 and 0.505, respectively (*P* < 0.05, *P* < 0.05). There was significant positive correlation between the amount of mandibular setback and the reduction in the transverse width of the glossopharynx with a coefficient of 0.364 (*P* < 0.05) (Table 6).

**Table 6:** Correlations between the amount of mandibular setback and the changes of the variables

Variable	Amount of mandibular setback(ΔB(*y*))	
	Pearson correlation	*P* value
Hyoid bone position measurements		
ΔHb(*x*)	0.109	0.566
ΔHb(*y*)	0.410	0.024*
ΔHb(*z*)	0.505	0.004**
Tongue position measurements		
Palatal height	− 0.101	0.597
Intraoral airway volume	− 0.159	0.402
PNS_perp_-TT/PNS_perp_-U1	0.182	0.337
Airway space measurements		
VP volume	− 0.128	0.501
GP volume	− 0.131	0.490
VP MCA	0.027	0.889
GP MCA	− 0.022	0.909
APW at VP	0.054	0.776
TRW at VP	− 0.072	0.703
APW at GP	0.243	0.196
TRW at GP	0.364	0.048*

The Pearson correlation coefficient was calculated to investigate correlations between the amount of mandibular setback and the changes of the hyoid bone, tongue, and oropharyngeal airway space. Amount of mandibular setback meant the change in *y* coordinate of B-point; ΔB(*y*). *VP* velopharynx, *GP* glossopharynx, *MCA* minimal cross-sectional area, *APW* anteroposterior width, *TRW* transverse width

*P < 0.05; **P < 0.01

## Discussion

In this study, the hyoid bone showed a posterior displacement with significantly increased values in the *y* coordinates. In addition, an inferior movement was observed with significantly decreased value in the *z* coordinate of the hyoid bone. This is consistent with the findings in other studies [3, 18], showing that this movement is an adaptation that prevents tongue encroachment into the pharyngeal airway. Also, Wickwire et al. [19] found that inferior movement of the hyoid bone after mandibular setback surgery was a physiological reflex for airway space maintenance. The tongue and hyoid bone are directly connected to the distal segment of the mandible at BSSRO by muscles such as the genioglossus muscle, geniohyoid muscle, and mylohyoid muscle. Mandibular setback leads to backward displacement of the distal segment, a condition that can result in change of the hyoid bone position.

Regarding changes in the tongue, significant positional changes were recorded at T1. Postoperatively, the dorsal surface of the tongue moved upward to the palatal side and the tip of the tongue moved backward, which is associated with the posterior and upright positioning of the tongue due to narrowing of the oral cavity after mandibular setback osteotomy. But dimensional changes were not significant. Achilleos et al. [20] found increased tongue length at the 3-year follow-up after mandibular setback osteotomy, but they reported no significant differences in tongue length and height between pre-surgery and about 6 months after surgery. The result of our study could be due to the relatively short follow-up period.

Since the structures of the hyoid bone and tongue are directly related to each other, the displacement of hyoid bone serves as an indicator of tongue position. Therefore, changes in the tongue position can also be assessed by measuring changes in the hyoid bone position. The long-term effects of bimaxillary surgery on the hyoid bone have been evaluated in several studies. However, few studies have compared mandibular setback surgery in class III patients. Aydemir et al. [21] reported no significant differences in the position of the hyoid bone between bimaxillary and mandibular setback surgery groups during a 1-year follow-up period. Efendiyeva et al. [22] showed significant superior movement of the hyoid bone after bimaxillary surgery. The authors also found adaptation had occurred to the normal position in the 5-year follow-up. So far, there has been no consensus regarding the positional changes of the hyoid bone after different class III orthognathic surgeries.

The pharyngeal airway is an anatomical space divided into the nasopharynx, oropharynx, and hypopharynx. These spaces are separated by soft palate and the upper top of epiglottis cartilage from the upper to lower end, but there is no consensus in the literature for an established standard of airway segmentation [23]. According to a meta-analysis published by Mattos et al. [24], there is moderate evidence to conclude that mandibular setback surgery may cause a reduction in the oropharyngeal airway. Therefore, the present study specified the area of interest as an oropharynx divided into upper and lower parts, using the end of the soft palate and tip of the epiglottis as landmarks.

With regard to changes in the oropharyngeal airway space, volumes and minimal cross-sectional areas of the velopharynx and glossopharynx significantly decreased at T1. Also, significant decreases in the anteroposterior and transverse width were observed at the MCA of the velopharynx and glossopharynx. These results are consistent with findings by Hong et al. [25]. The authors examined CBCT scans before surgery and 2 months after surgery and reported that the anteroposterior dimensions, cross-sectional areas, and pharyngeal airway volumes had all decreased in patients who had had mandibular setback surgery.

Because mandibular prognathism can be corrected by bimaxillary surgery or mandibular setback surgery, the two types of orthognathic surgery were compared most frequently in the same study. In a recent review, Christovam et al. [26] concluded that there is moderate evidence that infers the total volume of the upper airway decreases significantly after bimaxillary and mandibular setback surgery. Also, the authors mentioned that although the volume of the upper airway decreased less with bimaxillary surgery than in the mandibular setback alone, the treatment effect was not significant.

A pilot study was conducted to investigate the possible effects of genioplasty. We calculated both the amount of setback and the positional changes of the menton to segregate the possible effects of the genioplasty [17]. According to our pilot study results, there were no significant differences in the hyoid bone, tongue, and oropharyngeal airway space regardless of whether genioplasty was performed or not. This may be due to the deficient amount of chin displacement which was not significant enough to affect the muscles attached to the genial tubercle. Therefore, this study included patients who had undergone genioplasty at the same time.

Correlation analysis for this study showed some significant positive correlations between the amount of mandibular setback and the amount of posterior and inferior movement of the hyoid bone or the reduction in the transverse width of the glossopharynx, respectively, but not strong correlations. Similar to our findings, in a study conducted by Kawamata et al. [2], downward and backward displacement of the hyoid bone was seen postoperatively. The authors also reported that there were positive correlations between the amount of mandibular setback and reduction in the lateral width of the pharyngeal airway or the amount of hyoid bone displacement. Irani et al. [27] reported some significant correlations between mandibular posterior displacement and pharyngeal airway volumes or dimensions, but the authors suggested that the correlations were weak.

In clinical situations, it is important to understand the changes in the hyoid bone, tongue, and pharyngeal airway space after mandibular setback surgery. This is because the posterior movement of the mandible accompanies the changes of the hard and soft tissue, which are related to postoperative stability as well as to the postoperative obstructive sleep apnea (OSA). Therefore, when establishing a treatment plan for mandibular setback surgery, not only skeletal changes but also changes in surrounding structures such as the hyoid bone, tongue, and pharyngeal airway space should be considered. In addition, special attention should be paid to patients with obesity, short necks, macroglossia, large uvulas, and excessive soft tissue around the nasopharyngeal region, conditions which are associated with obstructive sleep apnea [28]. For such patients, changes in the surgical plan should be considered when necessary.

In this study, the early 1-month follow-up postoperative examination (T1) might be considered too soon to evaluate long-term PAS changes. While this examination was originally performed for surgical purposes to observe postoperative soft tissue edema, it provided an opportunity to analyze the surgery’s impact on the measurements.

Long-term follow-up (T2) was possible in 7 of the study subjects. The mean postoperative period was 13.83 months (range, 7.93–19.87 months). TGL and PNS_perp_-TT/PNS_perp_-U1 were significantly increased at T2, but the other variables were not significantly different. More samples are needed to better understand this relapse pattern.

Unfortunately, this study had some limitations. This study could not have polysomnography (PSG) recordings of the subjects. The sleep parameters in PSG consist of diverse variables related to respiratory function and sleep quality. In the future, it is necessary to use computed tomography to find out the correlations between changes in sleep parameters in PSG and 3D changes in the pharyngeal airway dimension. Also, the sample was relatively small and the assessment lasted for only a relatively short period. Further studies with a larger sample size are needed to identify the long-term changes relative to respiration function. Additionally, patients who underwent genioplasty were included in the study but were not divided into various directions of chin segment movement. Thus, the effects of genioplasty on changes in anatomical structures could not be accurately quantified. Further studies with a larger sample size are needed to determine the possible effects of genioplasty and to identify the long-term changes in consideration of a proper justification for a solid and optimized CBCT protocol.

## Conclusions

Even though the follow-up period was short in the present study, we were able to examine the changes in the hyoid bone, tongue, and oropharyngeal airway space with CBCT images after mandibular setback surgery. From this study, we observed the following:

1. The hyoid bone moved backward and downward.
2. The dorsal surface of the tongue moved upward, and the tip of the tongue moved backward.
3. There were significant decreases in the oropharyngeal airway space.
4. There were some significant, positive correlations between the amount of mandibular setback and the amount of posterior and inferior movement of the hyoid bone.

For successful results when combining surgical and orthodontic treatment, careful and precise treatment plans based on three-dimensional diagnostic information about hard and soft tissue structures are needed, including upper airway space. Clinicians should take into consideration the surgical method of choice, especially in cases which are associated with obstructive sleep apnea.

## Data Availability

The datasets used during the current study are available from the corresponding author on reasonable request.

## Abbreviations

3D: 3-dimensional
CBCT: Cone-beam computed tomography
PAS: Pharyngeal airway space
OSA: Obstructive sleep apnea
MCA: Minimal cross-sectional area
